# Transparency of the environmental cost – benefits of research? The “ADESC – Academic Environmental SCore” for publication

**DOI:** 10.1265/ehpm.25-00409

**Published:** 2026-01-24

**Authors:** Alexis Descatha, Dominique Savary, Celine Schnebelen

**Affiliations:** 1 Univ Angers, CHU Angers, Inserm, Univ Rennes, EHESP, Irset (Institut de recherche en santé, environnement et travail) - UMR_S 1085 Ester, SFR ICAT, CAPTV Angers, France; 2 Indiana University School of Public Health, Bloomington, USA; 3 Prevention Federation, CHU Angers, France; 4 CHU Angers, Emergency Department, Angers, France

**Keywords:** Health, Research, Sustainability, Publication, Impact, Consequences, Cost

Dear Editors,

Due to the importance of environmental consideration [1, 2], it is reasonable to ask about the environmental cost and consequences of research in scientific publications. Indeed, even publications are becoming increasingly transparent [3] - Funding, conflicts of interest, Institutional board review/ethical committed, use of artificial intelligence, translation - are now clearly stated alongside the individual contributions of authors. However, research of new medical devices and all innovations should be transparent of their impact: negative cost for environment and positive consequences. Indeed, it is reasonable to prioritize the positive impacts on science and health. However, it is also important to consider the environmental and public health consequences, as outlined in the One-Health model. Researchers have a responsibility to communicate their findings in a way that considers the potential impact on their ecosystem, with the aim of maximizing positive outcomes and mitigating any negative effects. Raising positive impacts on science or health is warranted but so should environmental and public health consequences.

A potential initial step in this process could be a global self-evaluation by each corresponding author who submitted the research. In practice, without necessarily going so far as to display a precise carbon footprint assessment for each paper as for healthcare [4, 5], a global self-assessment of negative and positive consequences on environment could be considered.

Here, we suggest a categorical score that could be called “ADESC, Academic Environmental SCore” (Fig. 1): 4 categories (“**very high**” environmental cost in red, “**high**” in orange, “**low**” in light green, and “**very low**” in dark green), adding in free text a few points of environmental attention in the research: products and devices, artificial intelligence and computers, travel and conferences, animal conditions, human cost and working condition, etc. In addition, the potential environmental benefits and positive impact on environment should also be included in the score, following the same model: 4 categories (“**very low**” positive environmental impact in red, “**low**” in orange, “**high**” in light green, and “**very high**” based on public health impact, workers, large population, environment and One health), with free text.

**Fig. 1:**
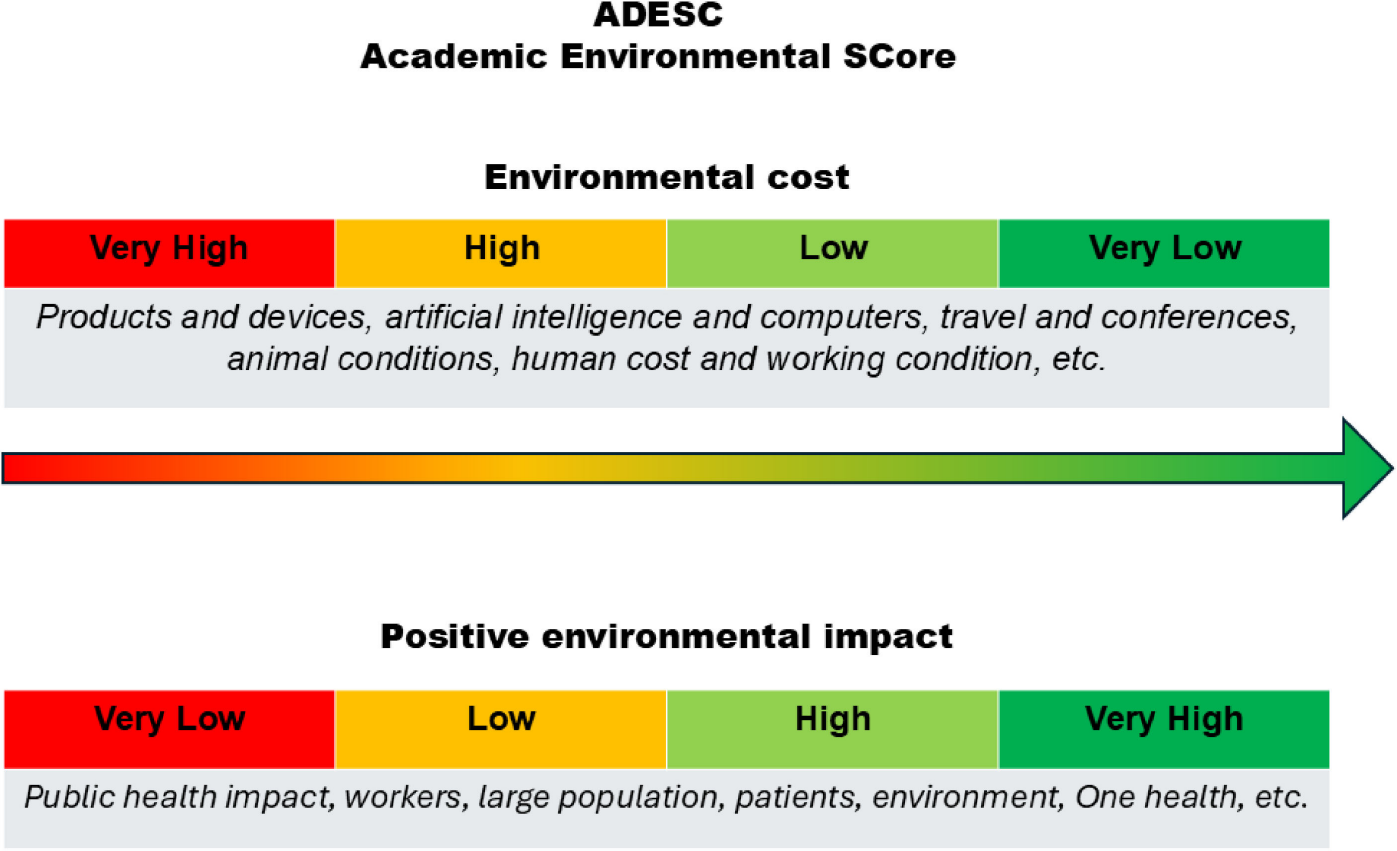
ADESC, Academic Environmental SCore

In the example this correspondence, we have quoted a “very low” environment cost (person’s opinion, computer with word processing software, Internet, and translation), and “**high**” positive environmental impact (launching a virtuous circle, though might take time, Table 1). Various virtual examples were presented, exhibiting “very high” financial costs but “very high” positive environmental impacts (new formulations of drugs oral instead intravenous), “high” financial costs and “very low” positive environmental impacts (large population data with artificial intelligence for the prognosis of one disease, Table 1).

**Table 1:**
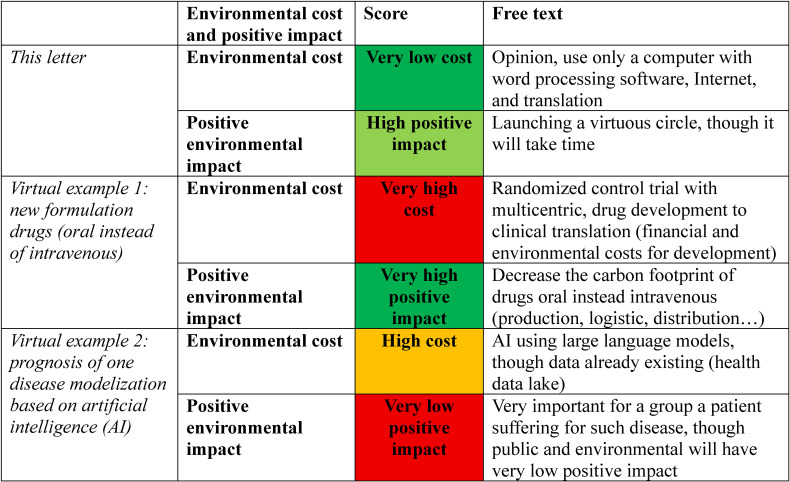
Quotation of ADESC for this letter (ADESC, AcaDemic Environmental SCore), and two virtual examples.

At this stage, the objective is to prioritize consideration of the ecosystem impact of research over the ADESC itself. It is not pertinent to draw comparisons between studies, ADESC colors can only be used to map studies in a specific field. Instead, the focus should be on initiating a virtuous cycle of eco-responsible research. Formal indicators based on precise carbon footprint, human/animal satisfaction, or even on a monetary basis would likely discourage all researchers from assessing ADESC. However, if this approach through this score proves successful, precise indicators will be necessary. These indicators will be based on examples and comments that justify the ADESC. It is important to mention that even very high cost/low positive impact should be continued if it helps patients.

In conclusion, ADESC would initiate a positive virtuous cycle for improving environmental considerations in medical research to improve sustainability. If the aimed cycle of eco-responsible research is launched, more precise indicators would need to be developed.

## Declarations

### Availability of data and materials

Not relevant.
